# Editorial: Integrin Ligands and Their Bioconjugate Systems: Synthesis, Conformation, and Biological Activity

**DOI:** 10.3389/fchem.2022.954618

**Published:** 2022-06-16

**Authors:** Rossella De Marco, Ali Dehshahri, Monica Baiula, Veronica I. Dodero

**Affiliations:** ^1^ Organic Chemistry, Department of Agricultural, Food, Environmental and Animal Sciences (Di4A), University of Udine, Udine, Italy; ^2^ Department of Pharmaceutical Biotechnology, School of Pharmacy, Shiraz University of Medical Sciences, Shiraz, Iran; ^3^ Department of Pharmacy and Biotechnology, University of Bologna, Bologna, Italy; ^4^ Organic, Biomolecular and Translational Chemistry, Department of Chemistry, University of Bielefeld, Bielefeld, Germany

**Keywords:** heterocycles, peptidomimetics, antagonists, nanoparticles, integrin, ligands, SAR

Integrins are one of the most important families of cell adhesion receptors that mediate cell-cell and cell-extracellular matrix interactions. These receptors are involved in tissue integrity and cell trafficking, growth, differentiation, proliferation, migration, and signal transduction. Integrins are also involved in pathological processes such as inflammation, wound healing, angiogenesis, and tumor metastasis. Therefore, pharmacological inhibition of integrins is of great interest for the treatment and prevention of diseases. The synthesis of antagonists capable of blocking the interaction of integrin with their cell adhesion molecule (CAM) or extra cellular matrix (ECM) ligands may represent a promising approach to treat various pathological conditions.

Integrin ligands, such as RDG, LDV, or BIO1211 show high activity *in vitro* but rapid enzymatic hydrolysis and clearance *in vivo*. Mimetics with unnatural amino acids or heterocycles and small molecule moieties have been developed in order to increase the metabolic stability of the ligands. A major unresolved issue in the development of new integrin-antagonists is their low level of activity compared to the precursor (e.g.: RGD or BIO1211). In this issue, a series of promising approaches for the conjugation of integrin ligands and their biological applications have been described.

Paulus and Sewald prepared a linear RGD mimetic containing small molecule-drug conjugate (SMDC). To prepare these mimetics the authors used an interesting approach namely the dual activity-difference (DAD) mapping, a methodology to visualize activity/selectivity changes against two different receptors upon partial structural changes in an array of molecules. They synthesized an RGD linear peptide and linked it by click-chemistry with an antimitotic agent monomethylauristatin E (MMAE) as SMDC. In parallel, the authors prepared cRGDfK-SMDC as a positive control, and cRADfK-SMDC as a negative control. The activity was tested by cell adhesion assay and the results showed that the RGD- and cRGDfK-SMDC displayed a micromolar potency to inhibit α_v_β_3_ integrin-mediated cell adhesion compared to the negative control which showed no effect.

As a different strategy, integrin ligands can also be exploited as targeting moieties, binding to overexpressed integrins on cancer cells in order to selectively deliver cytotoxic drugs. Schneider et al. described the development and evaluation of a promising targeted antitumor strategy. They used a scaffold composed of a human Fc functionalized by dextran, RGD ligands, and the cytotoxic drug MMAE. The Fc fragment was the centerpiece of the structure and was conjugated with both MMAE, through a self-immolative linker, and dextran, which allowed the multimeric presentation of RGD ligands. It was demonstrated that the Fc-dextran RGD-decorated scaffold was able to specifically bind to cancer cells endogenously expressing α_v_β_3_ integrin, if compared to a RAD-decorated scaffold, considered as a negative control. Moreover, RGD-decorated construct showed an anti-proliferative effect in cancer cells *in vitro*, suggesting integrin-mediated endocytosis, followed by endosomal cleavage of the MMAE linker and its subsequent release within cancer cells expressing α_v_β_3_ integrin.

Conjugation of integrin ligands to NPs represents an effective approach to address the intrinsic drawbacks of the native peptides, providing access to a variety of biomedical uses such as nano-carriers and drug delivery systems. Sacchi et al. explored this last approach. They used gold nanoparticles functionalized with cyclized CGisoDGRG peptide (*iso1*), which contains the *iso*DGR sequence to recognize α_v_β_3_ integrin receptor in cancer cells. *Iso1* was coupled via its thiol group to maleimide-PEG_11_-lipoamide (LPA) to obtain nanogold-*iso1*-PEG_11_-LPA. Nanogold-*iso1* can be functionalized also with bioactive tumor necrosis factor-α (TNF-α) and it can be stored for days or for longer than 1 year at low temperature without losing the capability to bind α_v_β_3_ integrin and TNF-α-mediated cytotoxic activity. In *in vitro* experiment, nanogold-*iso1*-PEG_11_-LPA demonstrated the ability to bind purified α_v_β_3_ and to α_v_β_3_-expressing cells. The activity of nanogold linked with both *iso1* and TNF-α was higher in removing the tumor in WEHI-164 fibrosarcoma-bearing mice than nanoparticles lacking cyclized CGisoDGRG peptide.

Besides targeted drug delivery, integrin ligands-conjugated molecules can also be used for tumor imaging. The biodistribution of imaging probes *in vivo* depends both on their chemical structure and their pharmacokinetic properties. The latter can be altered using different strategies, including the exploitation of serum albumin as a transporter. Holtke et al. described the modification of a previously developed fluorescent RGD mimetic probe with three different albumin binding moieties (ABMs), with the aim of improving its pharmacokinetic properties. In particular, α_v_β_3_-targeted fluorescent RGD mimetics biodistribution and tumor targeting abilities were analyzed for 1 week in a murine U87MG glioblastoma xenograft model. The results of the study showed that RGD mimetics modified with ABMs accumulated in tumor tissue for at least 5 days as compared to the unmodified probe, which was rapidly excreted. Moreover, the three ABM-modified probes displayed different biodistribution and excretion properties, thus allowing a fine-tuning of ABM modified RGD mimetics’ pharmacokinetic properties.

With this special issue, we collected some studies on new integrin ligands and their conjugated forms, and explored recent applications to help the development of new therapeutic strategies for diseases such as cancer.

